# Menopause and development of Alzheimer’s disease: Roles of neural glucose metabolism and Wnt signaling

**DOI:** 10.3389/fendo.2022.1021796

**Published:** 2022-10-19

**Authors:** Paulina Villaseca, Pedro Cisternas, Nibaldo C. Inestrosa

**Affiliations:** ^1^ Centro de Excelencia en Biomedicina de Magallanes (CEBIMA), Universidad de Magallanes, Punta Arenas, Chile; ^2^ Instituto de Ciencias de la Salud, Universidad de O´Higgins, Rancagua, Chile; ^3^ Centro de Envejecimiento y Regeneración (CARE UC), Facultad de Ciencias Biológicas, Pontificia Universidad Católica de Chile, Santiago, Chile

**Keywords:** wnt signaling, menopause, estrogen, glucose brain metabolism, alzheimer´s disease, tau phosphorylation, APP - amyloid precursor protein

## Abstract

Late onset Alzheimer´s disease (AD) is a neurodegenerative disease with gender differences in its onset and progression, being the prevalence predominant in women and at an earlier age than in men. The pathophysiology of the menopausal condition has been associated to this dementia, playing major roles regarding both endocrine and glucose metabolism changes, amongst other mechanisms. In the current review we address the role of estrogen deficiency in the processes involved in the development of AD, including amyloid precursor protein (APP) processing to form senile plaques, Tau phosphorylation forming neurofibrillary tangles, Wnt signaling and AD neuropathology, the role of glucose brain metabolism, Wnt signaling and glucose transport in the brain, and our research contribution to these topics.

## Introduction

The endocrine status that comes with menopause, with the cessation of ovarian estrogens and progesterone synthesis and increase of gonadotropins (FSH and LH), brings a loss of neuroprotector mechanisms that could explain cognitive decline (1) and the risk of developing AD later in life more prevalent in women (2). Estradiol (E_2_), the main estrogen acting during women´s reproductive years, is well known to exert neuroprotection through various mechanisms (3–5). Even more, neuron derived E_2_ regulates synaptic plasticity and memory (6, 7).

On the other hand, it is also well known that Wnt signaling plays important roles in various systemic physiological processes: cell differentiation, polarity and migration (8, 9). At the central nervous system (CNS) as well, Wnt signaling has been described in various cell types including radial glia, oligodendrocytes, microglia, astrocytes, and neurons (10–12), and regulates neural patterning, stem cells proliferation and neurogenesis (9, 13, 14). Back in 2000, we proposed that Wnt signaling might play a key role in AD neuroprotection (15). We have since studied the function of this signaling pathway in the CNS and its importance in AD, with the objective to explore pharmacological strategies that could strike the burden of this disease. In humans, 19 Wnt ligands and 10 Frizzled (Fz) receptors are recognized (16). Wnt proteins are post-translationally palmitoylated to be secreted and to be bound to Fz receptor (17). Wnt ligands play a key role in the formation and function of synapses through lifetime. Wnt7a, a canonical ligand, stimulates dendritic spine morphogenesis inducing the postsynaptic density-95 (PSD-95) protein expression (18).

Dysfunction of Wnt signaling has been described in aging and menopause, both associated with hippocampal memory impairments (9, 19). Our studies on hippocampal cultured neurons, as well as in transgenic mice models of AD, indicated that Wnt signaling protects against the amyloid β (αβ) peptide neurotoxicity. Wnt signaling is also involved in Tau phosphorylation by its enzyme glycogen synthase kinase-3β (GSK-3β) and, finally, the activation of Wnt signaling was shown to be involved in learning and memory (20–22).

One of the mechanisms through which E_2_ protects from brain tissue ischemia is by interfering the induction of dickkopf-1 (Dkk1) (23), an antagonist of the Wnt signaling pathway (24). Studies with hippocampal neurons in culture showed that Dkk1 is required for amyloid-mediated synaptic loss (25). Dkk1, though crucially expressed during neurodevelopment, its elevation in adult life has shown to be a principal mediator of neurodegeneration (9, 19).

Another mechanism related to a decrease in brain function of AD patients is the decrease in glucose consumption. Our research group described that Wnt activators increase both brain glucose consumption and cognitive performance in transgenic AD mice (26).

A Wnt activator Andrographolide (ANDRO), a labdane diterpene, was found to reduce Aβ burden, astrogliosis, interleukin-6 and oxidative stress (27), all these mechanisms present in AD. Our studies are consistent with the idea that the activation of Wnt signaling might delay the onset of AD (9).

In this review, we describe studies on the role of Wnt signaling on the neuroprotective activity of E_2_, and on energy metabolism and glucose transport in neurons, in search of a method that could delay or avoid the development of this progressive dementia with no effective treatment available.

## Menopause and development of AD

A particularly interesting aspect is the role of sex steroids on neuroprotection (28). In fact, postmenopausal women are most frequently affected by sporadic AD in a ratio 3:1, differently from the incidence in the infrequent hereditary illness occurring in younger patients in a ratio 1:1 (29). Estrogen deficiency occurring in spontaneous or surgical menopause has been associated to cognitive decline; in this context, several studies have shown that early E_2_ replacement exerts neuroprotective effects (7, 30).

Since AD is the most prevalent dementia and incurable disease, understanding its molecular pathogenesis could lead to the development of a specific therapeutic strategy, here we will describe effects of E_2_ on Aβ peptide production and Tau phosphorylation.

### E_2_ affects the production of Aβ peptide by blocking the amyloid precursor protein processing

The APP is the precursor of a 40-42 amino acid peptide called amyloid-β-peptide (Aβ), that forms the central core of senile plaques (31). APP is cleaved by distinctive enzymatic activities, denominated secretases: *α-secretase* was the first described and cuts the Aβ peptide between the 15 and 17 amino acids and thus yields a large soluble amino-terminal fragment of APP known as the soluble non-amyloidogenic APP (sAPP), that does not form senile plaques. The second enzyme, named *β-secretase* (BACE1), cuts the Aβ peptide at its N-terminal domain, generating a cell-associated amyloidogenic carboxy-terminal fragment which is processed by a third enzymatic complex enzyme called *γ-secretase*, which produces a soluble Aβ by cutting the APP in the middle of the neuronal membrane, generating an aggregating amyloidogenic Aβ peptide that forms the senile plaques (31).

One of the first indications that E_2_ was protective against AD was obtained when E_2_ was found to stimulate the processing of APP, at the level of the *α-secretase* enzyme, preventing the generation of Aβ, generating a soluble non-amyloidogenic APP (32); therefore, E_2_ blocks the amyloid plaques formation (32), a key neuropathological lesion in AD (31). In addition, brain E_2_ deficiency accelerates amyloid plaque formation, as shown in an AD animal model (33). Finally, E_2_ also decreases the formation of amyloid fibrils as well as the formation of Aβ oligomers *in vitro* (34). **Table 1** indicates some of the E_2_ effects on Aβ peptide production.

**Table 1:** Summary of Neuroprotective actions of E_2_.

**At the level of Tau protein**
E_2_ prevents hyperphosphorylation of Tau protein (35)
E_2_ inhibits PHF-Tau conformation through miRNA 218 (36)
**At the level of Aβ protein**
E_2_ protects against neurotoxicty by Aβ (4, 37)
E_2_ stimulates the degradation of Aβ peptide (32, 38)
E_2_ decreases the formation of Aβ fibrils and Aβ oligomers (34, 39)

The number in each line corresponds to the references.

#### Wnt signaling activity and Aβ peptide production

Recent studies in mouse AD models indicate that the activation of the Wnt signaling pathway decreases the Aβ peptide levels, due to a low *β-secretase* (BACE1) availability, since Wnt signaling activation repressed BACE1 transcription at the nuclear level (40, 41). These results indicate that the activation of Wnt signaling by acting in a different secretase than E_2_, also decreases the amount of Aβ peptide and diminishes the amyloid plaques formation (9, 41). These studies also indicated that reducing the β-cleavage of APP may protect against the appearance of AD in the animal models. Interestingly, molecular genetics studies in Iceland (42) identified coding variants in the APP that were tested for an association with AD, after studying whole-genome sequencing of 1,795 subjects. Variant A673T, corresponding to a single nucleotide alanine-to-threonine substitution adjacent to the *β-secretase* (BACE1) site in APP, was markedly more common in the elderly control than in the AD subjects; this is consistent with an ≈ 40% reduction in the Aβ, formation observed *in vitro* (42).

### E_2_ affects Tau phosphorylation

Intraneuronal communication occurs through microtubules and the associated Tau proteins that stabilize microtubule structure and function. In AD, kinases phosphorylate Tau proteins, detaching them from the microtubules; these “speedways” lose structure and function, and synaptic vesicles cannot be driven to the synaptic region, and twisted filaments are aggregated in tangles – the neurofibrillary tangles. In fact, Tau is phosphorylated by several kinases, including GSK-3β (43). In general, GSK-3β can phosphorylate around 42 Tau sites, of which at least 29 are found phosphorylated in human AD brains (44).

In this context, it is interesting to mention that E_2_ prevents neural Tau hyperphosphorylation; moreover, E_2_ increased Tau dephosphorylation as measured by using a Tau-1 antibody which identifies a site of Tau (proline) which is non-phosphorylated. In addition, E_2_ prevented okadaic acid-induced hyperphosphorylation of Tau in both proline- and non-proline sites, and this effect was blocked by an anti-E_2_ antibody (45). In this study, the Estradiol Receptor (ER) appeared to be responsible of these effects probably *via* the Akt metabolic pathway (35). Finally, in transgenic mice expressing the wild-type human Tau, it was shown that E_2_ inhibited the Aβ-mediated conformation of Tau called paired helical filaments (PHF) through both antioxidant activity and regulation of the miRNA-218 (28, 46). **Table 1** indicates some effects of E_2_ on Tau protein.

#### Wnt signaling activity and Tau hyperphosphorylation

β-Catenin is a key protein of Wnt signaling which is regulated by GSK-3β, studies in ovariectomized rats sacrificed 1h after E_2_ treatment, showed that β-catenin and GSK-3β are co-immunoprecipitated with the (ERα) in the hippocampus. This observation is consistent with the hypothesis that a multi-complex is formed by ERα, β-catenin and GSK-3β, that inhibited GSK-3β activity and thus regulating Tau phosphorylation, avoiding its hyperphosphorylation through Wnt and estradiol action (44, 47).

AD mutations in presenilin-1 also promote GSK-3β activity and Tau phosphorylation (48). Overexpression of GSK-3β in the adult mouse brain leads to a decrease in β-catenin and an increase in Tau phosphorylation (45). Also, hippocampal infusion of Dkk1, a Wnt antagonist of the Canonical Wnt signaling triggers PHF1 Tau phosphorylation in rats (46).

### Energy metabolism and metabolic syndrome

The excess of energy from carbohydrates and fats in inadequate diet habits, leads to progressive metabolic disorders at any age. In the perimenopausal years, when E_2_ synthesis becomes variable and fluctuating, and FSH increases, the risk of the appearance of the metabolic syndrome increases significantly; when E_2_ decline is established with menopause, the metabolic syndrome is clearly higher than in premenopausal years (49). All components of metabolic syndrome become more conspicuous with menopause: higher blood glucose, lower HDL-cholesterol, higher blood triglycerides, higher blood pressure, and increase in visceral adiposity (larger waist circumference) (50).

In postmenopausal women, treatment with estrogens improves all components of the metabolic syndrome, as well as insulin resistance parameters (51). Estrogens have a transcendent role on metabolism by modulating directly whole-body energy management, controlling glucose availability and facilitating insulin secretion, and modulating energy partition by favoring lipids as the main substrate for energy when they are more available than carbohydrates, shifting from lipid storage to their oxidation as substrate. As well, E_2_ maintains energy balance by influencing energy intake and energy expenditure, regulating body weight homeostasis. The E_2_ roles on energy balance are mediated by the (ERα), which is abundant in the hypothalamus. Estrogen deficiency in menopause and the consequent loss of ERα activity, determine a decrease in energy expenditure, increased food intake and increased adiposity (52–54). In animal experiments, treatment with E_2_ decreases obesity (55), decreases hepatic steatosis, and limits fat deposition (54, 56).

Estrogens, as well, decrease lipogenesis and inhibit adipogenesis through ERα activation (57). The role of E_2_ on lipid metabolism is coordinated with effects on carbohydrate metabolism, also through ERα, by lowering insulin resistance and fat storage (58, 59). The insulin-sensitizing actions of E_2_ work through improving insulin-mediated glucose uptake, insulin signaling, and glucose transport in adipose. muscular and brain tissue. E_2_ also controls the metabolic sensor in the hypothalamus, protecting the brain from hypoglycemia (60). Otherwise, E_2_ potentiates the oxidative capacity of mitochondria and E_2_ deprivation induces mitochondrial dysfunction and insulin resistance, mechanisms involved in the development of alterations in cognitive function (61). Thus, the metabolic changes associated to menopause can play a key role in triggering AD, in addition to the direct mechanisms favoring the Aβ peptide production of amyloid plaques and the neurofibrillary tangles described above.

The role of glucose metabolism in the brain and its regulation by Wnt signaling is discussed now.

## Glucose metabolism in the brain

The adult brain represents only 2% of the total body mass but is responsible for the utilization of almost 25% of total ATP produced by the body (62–64). Glucose is the principal energy source of the brain (65, 66). The uptake of glucose occurs *via* glucose transporters (GLUTs): these are 14 isoforms, and several GLUTs are expressed in the brain. At cellular levels the specific isoform expressed depends on the brain region, i.e., in the cortex, astrocytes express exclusively GLUT1 while neurons express mainly GLUT3 (67–69). In the hippocampus the expression of mRNA of GLUT4 mainly occurs in neurons; this is an interesting observation because GLUT4 is regulated by insulin and AMP-activated protein kinase (AMPK) pathways (70, 71). In different AD models, a significant decrease in the expression of GLUT4 has been described, suggesting a role for this transporter in AD pathogenesis (72).

After glucose uptake by the cells, this molecule can be used by several pathways including glycolysis, the Krebs cycle and oxidative phosphorylation, the pentose phosphate pathway (PPP) and glycogen synthesis (73–75). The use of glucose by glycolysis, together with the Krebs cycle are the major source of ATP to neurons (76–78). The PPP is required to obtain reducing equivalents in the form of nicotinamide adenine dinucleotide phosphate (NADP(H^+^)), which in turn is required for neuronal defense against oxidative stress since it is necessary for the recycling of antioxidant molecules, such as glutathione and ascorbate (74, 79). The glucose utilization by brain cells plays a central role in the physiology of the brain and a decrease in this metabolism has been related with almost all the neurodegenerative diseases, including AD (62).

### Glucose metabolism in AD

A decrease in glucose utilization has been described in several brain regions of AD patients, mainly in brain zones related functions such as memory/learning, including the hippocampus and cortex (62, 72, 80). At the molecular level, the lower glucose utilization in AD patients has been associated with alterations, including decreased expression of GLUT1, -3 and -4 in the cortex and hippocampus, insulin resistance in the brain, mitochondrial dysfunction, deregulation of the Krebs cycle, and oxidative phosphorylation, which are triggered by the loss of key enzymes (81–83). On the other hand, stimulating glucose metabolism in AD patients through the administration of insulin or GLP-1, significantly improves the cognitive function, supporting the close relationship between the deregulation of cerebral glucose metabolism and the cognitive failures described in AD (84, 85). In the context of menopause, hot flashes could be a neurovascular compensatory response to brain hypometabolism, to increase blood flow and glucose in the brain, as has been shown in perimenopausal women (86).

### Wnt signaling and glucose metabolism

Wnt ligands have been related with the regulation of glucose metabolism. Animal models and human studies have suggested that some components of Wnt signaling increase the risk for the development of diabetes and age-related dementia (9, 87).

The increase in Ca^+2^ levels by the Wnt signaling pathway significantly affects glucose metabolism in neurons and astrocytes; thus, Wnt/Ca^2+^ signaling may represent a newly identified mode for the regulation of glucose metabolism in neurons (88–90). Previously, we showed that acute treatment with Wnt5a stimulates glucose uptake in neurons, in a time-dependent manner, this was correlated with an increase in both hexokinase activity and the glycolytic rate. Furthermore, we observed an increase in the activity of glucose-6-phosphate dehydrogenase and PPP. The effects of Wnt5a were dependent on the generation of nitric oxide (NO) downstream of Wnt5a signaling (62, 91). These results support that the activation of non-Canonical Wnt signaling pathway regulates cellular glucose metabolism in neurons in a NO-dependent manner (74, 92).

When we treated the neurons with a canonical Wnt ligand, like Wnt3a, we observed an increase in glucose uptake in neurons without changing the expression or localization of GLUT3, the main GLUT transporter in these cells. Furthermore, we described that the acute treatment with Wnt3a stimulates the activation of Akt, the activity of hexokinase and the glycolytic rate. These effects of Wnt3a were dependent on activation of the Akt pathway and was independent of both the transcription of Wnt target genes and synaptic effects (93).

In addition to Wnt ligands, other molecules such as ANDRO, obtained from *Andrographis paniculata*, activates the Wnt signaling pathway by inhibiting GSK-3β and it was shown to protect neurons (94, 95). ANDRO also increases the uptake of glucose *in vivo* and *in vitro* promoting in both conditions a recovery of the brain glucose metabolism and cognitive performance (9, 26, 96). Furthermore, we also demonstrated in a transgenic mice model of AD that the administration of ANDRO in pre-symptomatic stages can delay the appearance of several markers of AD, promoting a general rescue of the brain metabolic parameters (9, 62, 97).

These studies support that Wnt signaling promote glucose metabolism in neurons, stimulating the ATP production in these cells to satisfy the energy demands of neurons (62, 91, 93).

**Figure 1:**
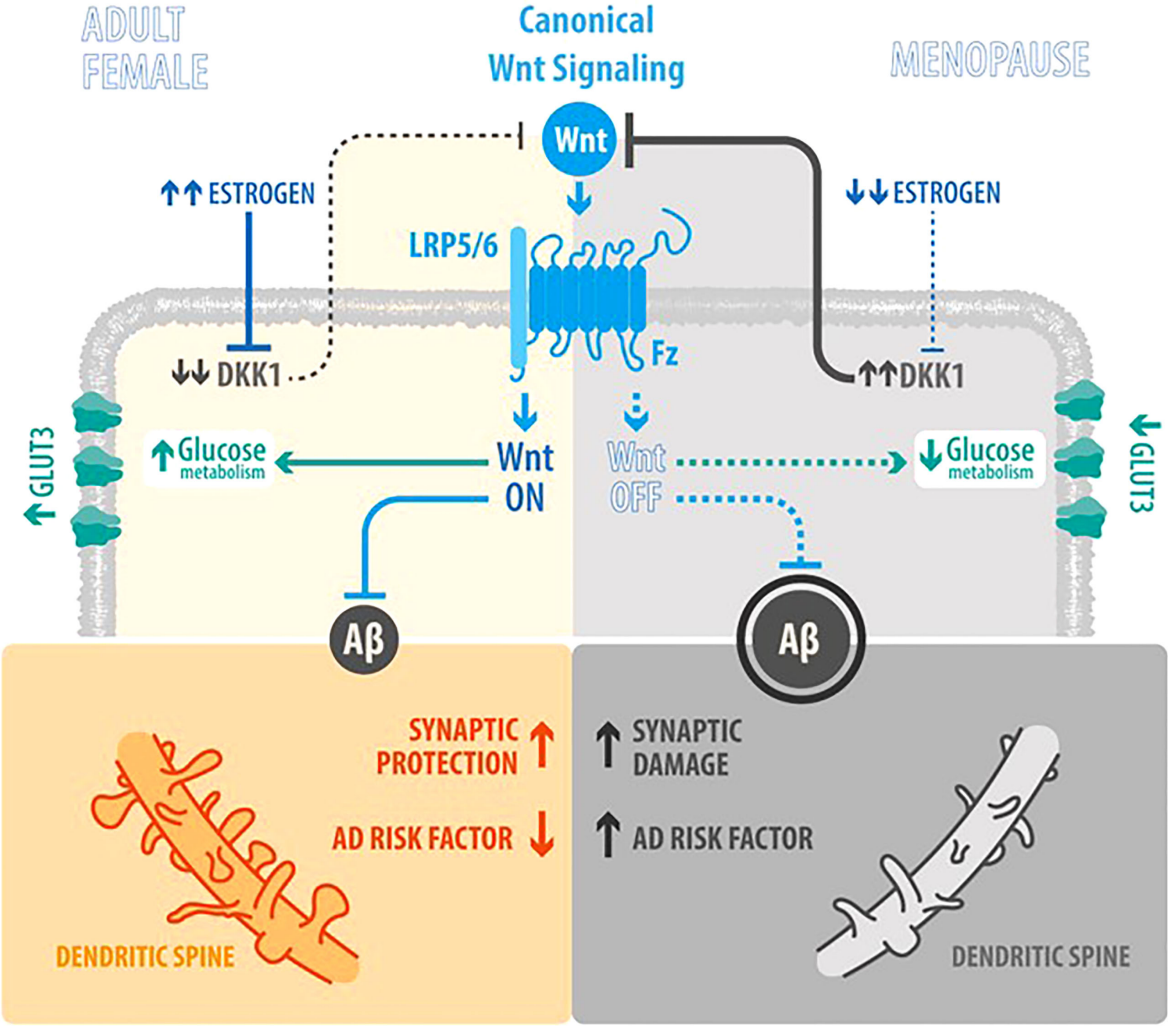
How factors are interrelated and their role in AD (Estrogen, Wnt signaling, glucose metabolism and neurodegeneration). Scheme integrating the actions of Wnt signaling, Estrogen levels, Aβ peptide synthesis and brain glucose uptake in the Adult Female as opposed to the Menopause status: *In the Adult Female*, estrogen (E_2_) inhibits the synthesis of Dkk1 (a physiological antagonist of Wnt canonical signaling) (23), thus promoting the activation of Wnt canonical signaling and leading to a decrease of Aβ peptide synthesis, an increase in synaptic protection and development of dendritic spines; in parallel, also leading to increased brain glucose uptake. *In Menopause*, the decrease of E_2_ induces an increase of Dkk1 and the consequent decrease in Wnt signaling activity, that lead to an increase and aggregation of Aβ peptide and neuronal damage, as the loss of dendritic spines by the accumulation of plaques; in parallel, the decrease in Wnt signaling activity, decreases brain glucose metabolism.

Globally, estrogens play a role on neurodegeneration interrelated with Wnt signaling and glucose brain metabolism as described in **Figure 1**, a scheme comparing the estrogenic adult female with the postmenopauseal estrogen-deprived brain.

A strength of this review is the involvement of our group in a lifetime research on this topic, beginning in the early 1998 (3, 4) and ongoing research (9).

## Conclusion

Our aim to integrate the actions of different factors: Wnt signaling, E_2_ levels, synthesis of Aβ peptide, Tau phosphorylation and brain glucose uptake have been detailed in this contribution, and are intimately entwined for neuroprotection in the female reproductive years. These mechanims are lost with menopause leading to a cascade of events towards neurodegeneration in the postmenopausal years.

Further research is required for a better understanding of the cellular and physio-pathological mechanisms related to the endocrine and metabolic changes that occur with menopause, globally and in the brain. This could lead to discover triggering factors that initiate late onset neurodegeneration and that facilitate its development. Thence, science could be nearer to intervene and strike on this disease burden in an aging population worldwide.

## Author contributions

PV and NI designed the review. PV, PC and NI wrote the article and edited the article. NI prepared **Table 1**. PC prepared **Figure 1** with graphic designer Felipe Serrano. All authors contributed to the article and approved the submitted version.

## Funding

This work was supported by grants from the Basal Centre of Excellence in Aging and Regeneration AFB170005 and ACE210009 from ANID (Agencia Nacional de Investigación y Desarrollo of Chile) to NCI.

## Acknowledgments

We thank to Illustrative Science (www.illustrative-science.com), for the Illustrated Graphic of **Figure 1**.

## Conflict of interest

The authors declare that the research was conducted in the absence of any commercial or financial relationships that could be construed as a potential conflict of interest.

## Publisher’s note

All claims expressed in this article are solely those of the authors and do not necessarily represent those of their affiliated organizations, or those of the publisher, the editors and the reviewers. Any product that may be evaluated in this article, or claim that may be made by its manufacturer, is not guaranteed or endorsed by the publisher.
